# Detecting intergene correlation changes in microarray analysis: a new approach to gene selection

**DOI:** 10.1186/1471-2105-10-20

**Published:** 2009-01-15

**Authors:** Rui Hu, Xing Qiu, Galina Glazko, Lev Klebanov, Andrei Yakovlev

**Affiliations:** 1 Department of Biostatistics and Computational Biology, University of Rochester, 601 Elmwood Avenue, Box 630, Rochester, New York 14642, USA; 2 Department of Probability and Statistics, Charles University, Sokolovska 83, Prague 18675, Czech Republic

## Abstract

**Background:**

Microarray technology is commonly used as a simple screening tool with a focus on selecting genes that exhibit extremely large differential expressions between different phenotypes. It lacks the ability to select genes that change their relationships with other genes in different biological conditions (differentially correlated genes). We intend to enrich the above procedure by proposing a nonparametric selection procedure that selects differentially correlated genes.

**Results:**

Using both simulations and resampling techniques, we found that our procedure correctly detected genes that were not differentially expressed but differentially correlated. We also applied our procedure to a set of biological data and found some potentially important genes that were not selected by the traditional method.

**Discussion and Conclusion:**

Microarray technology yields multidimensional information on the function of the whole genome. Rather than treating intergene correlation as a nuisance to the traditional gene selection procedures which are essentially univariate, our method utilizes the rich information contained in the correlation as a new selection criterion. It can provide additional useful candidate genes for the biologists.

## Background

It has become common practice to use microarray technology to find "interesting" genes by comparing two or more different phenotypes. Modern methods of microarray data analysis typically employ two-sample statistical tests to test differential expression of genes, combined with multiple testing procedures to guard against Type 1 errors (see [1,2] for reviews). Such methods are biased towards selecting those genes that display the most pronounced differential expression. Once the list of genes showing statistically significant differential expression has been generated, these genes are often ranked using purely statistical criteria and this ranking is thought to reflect their relative importance. Quite typically, a certain number of genes with the smallest *p*-values are finally selected from the list of all "significant" genes. While most biologists recognize that the magnitude of differential expression does not necessarily indicate biological significance, in the absence of better methods, this remains the dominant means to initially prioritize candidate genes. From a biological perspective, the above-described paradigm is far from a perfectly valid approach, because genes are not independent entities – they can interact with each other in many ways. As an example, a "chain reaction" type of a dependence structure of gene expressions was documented in the literature [3]. In such situation, even a very small change in expression of a particular gene may have dramatic physiological consequences if the protein encoded by this gene plays a *catalytic *role in a specific cell function. Many other downstream genes may amplify the signal produced by this truly interesting gene, thereby increasing their chance to be selected by formal statistical methods. For an upstream regulatory gene, however, the chance of being selected by such methods may diminish as one keeps hunting for downstream genes that tend to show bigger changes in their expression. As a result, the initial list of candidates may be inflated with many effector genes that do little to elucidate the fundamental mechanisms of biological processes.

There are two natural ways to remedy this situation. One way is to use bioinformatics tools that utilize prior biological knowledge, such as partially known pathways, to prioritize candidate genes. This approach is now routinely used in biological studies and there are ongoing efforts to enrich it with specially designed algorithms [4]. The main weakness of the above approach is that the current biological knowledge is still quite limited and sometimes inaccurate. Another way is to extract additional information on the changes of the *relationships *between different genes from microarray data by using pertinent statistical methods.

For example, if an upstream gene ceases to be catalytic in one phenotype, or this gene is active in two *different *biological pathways in two phenotypes, a carefully designed statistical test based on the intergene dependence structure should be able to detect this change. In more general situations, intergene dependence structure alone may be insufficient to pick up those upstream genes directly, but knowing the relationship changes across conditions points out possible directions for searching the interesting genes.

Notwithstanding the importance of testing for differential expression of genes, we suggest approaching the problem of microarray data analysis from a different angle. We designed a new method to select those genes that are likely to change their relationships with other genes. More specifically, we suggest selecting candidate genes using a statistical test that detects changes in the whole correlation vector associated with each gene. This additional information will be instrumental in making the final selection of candidate genes more meaningful.

We propose to enrich the statistical inference from microarray gene expression data by testing the following hypothesis: *the ith gene does not change its relationships with all other genes across the two phenotypes (conditions) under study*. This can be accomplished by comparing the joint distribution of the correlation coefficients between this gene and other genes in different conditions.

We conducted a series of simulations with different configurations. The results obtained by our method were compared with those of a similarly designed univariate selection procedure. We observed that our method correctly selected those genes which change correlations with other genes but retained the same marginal distributions.

We also conducted various experiments with biological data. A large set of childhood leukemia data available from St. Jude Children's Research Hospital [5] were used. Our method selected some genes which were not selected by a comparable univariate approach.

## Biological data

The biological dataset used in this study was from the St. Jude Children's Research Hospital (SJCRH) Database on childhood leukemia [5]. Two groups of data were employed: patients (*n *= 88) with hyperdiploid acute lymphoblastic leukemia (**HYPERDIP**) and patients (*n *= 79) with a special translocation type of acute lymphoblastic leukemia(**TEL**). To make two data groups more comparable, only the first 79 patients in **HYPERDIP **were used.

Since the original probe set definitions in Affymetrix GeneChip data were known to be inaccurate [6], we updated them by using a custom CDF file to produce values of gene expressions. The CDF file was obtained from . After that, each patient was represented by an array reporting the logarithm (base 2) of expression level on the set of 7084 genes. For both datatsets, each gene was standardized so that it had zero mean and unit standard deviation. This was to avoid introducing false correlation coefficients when doing permutations.

## Methods

### Correlation vectors

Let us denote *m *as the number of genes. For the *i*th gene, we computed the (*m *- 1)-dimensional random vector **r**_*i *_= (*r*_*i*1_, ⋯, *r*_*i*, *i*-1_, *r*_*i*, *i*+1_, ⋯, *r*_*im*_). Here *r*_*ij *_is the sample correlation coefficient between the *i*th and the *j*th gene. This vector represents the relationships between the *i*th gene and all other genes. Denote the (*m *- 1)-dimensional joint distribution functions of **r**_*i *_in two different conditions by $F_{r_{i}(A)}$(*x*) and $F_{r_{i}(B)}$(*x*). A pertinent statistical test can be designed to test the basic null hypothesis

(1) $$H_{i}:F_{r_{i}(A)}(x)=F_{r_{i}(B)}(x).$$

To increase the sensitivity of our test to departures from **H**_*i*_, especially when the correlation coefficients are very high, we applied the Fisher transformation to the sample correlation coefficients:

(2) $$w_{ik}=\frac{1}{2}\log\frac{1+r_{ik}}{1-r_{ik}},$$

where *k *= 1, ⋯, *i *- 1, *i *+ 1, ⋯, *m*. The power improvement was confirmed by our simulation (see Table 1). We denote the *correlation vectors *in two conditions by **w**_*i*_(*A*) and **w**_*i*_(*B*), respectively.

**Table 1:** SIMU2, true positives (TP) and false positives (FP) in simulations with dependent base.

**Effect Size**	**CV Method**		**WRS Method**	
		
	FP mean(STD)	TP mean(STD)	FP mean(STD)	TP mean(STD)
**0.1**	0.25(0.7)	0.1(0.3)	0.05(0.22)	0.0(0.0)

**0.2**	0.9(2.39)	1.2(4.79)	0.15(0.48)	0.05(0.22)

**0.3**	1.1(3.51)	4.9(10.77)	0.15(0.48)	0.2(0.87)

**0.4**	0.9(3.48)	80.5(25.79)	0.65(1.35)	0.0(0.0)

In CV method with effect size 0.4, the TP drops to 2.9(6.82) without Fisher transformation.

Total number of genes: 708. Number of differentially correlated genes: 100. Method: group method. Extended Bonferroni threshold: 1.0.

Instead of testing **H**_*i*_, we tested

(3) $${H^{\prime }}_{i}=F_{w_{i}(A)}(x)=F_{w_{i}(B)}(x),$$

where $F_{w_{i}(A)}$(*x*) and $F_{w_{i}(B)}$(*x*) are the joint distribution functions of **w**_*i*_(*A*) and **w**_*i*_(*B*), respectively. If ${H^{\prime }}_{i}$ was rejected, we declared the *i*th gene to be a *differentially correlated *gene.

In order to test the hypotheses based on the joint distribution functions of correlation vectors, we needed to create samples of correlation vectors. The following two methods were employed for this purpose:

• Group method: Divide each dataset into 8 subgroups, each containing 10 slides (9 slides for the last subgroup of the biological data). By computing correlation vectors from each subgroup, we obtained a sample of size 8.

• Resampling method: Randomly select 60 slides to calculate the correlation vector. Repeat 20 times to get 20 correlation vectors in each group, respectively.

Through the simulations, we found that these two methods were comparable, with the resampling method being slightly better in terms of testing power (see Table 1, 2, 3 and 4). However, the resampling method was much more computationally demanding. As an example, it took approximately 30 hours to analyze the biological data (7084 genes and 79 slides in both conditions, 10,000 permutations) with the group method.

**Table 2:** SIMU1, true positives (TP) and false positives (FP) in simulations with independent base.

**Effect Size**	**CV Method**		**WRS Method**	
		
	FP mean(STD)	TP mean(STD)	FP mean(STD)	TP mean(STD)
**0.1**	1.0(1.0)	4.1(4.15)	0.65(0.85)	0.25(0.54)

**0.2**	0.6(0.73)	37.4(14.47)	0.75(0.83)	0.1(0.3)

**0.3**	1.1(1.04)	85.45(11.1)	0.9(1.09)	0.1(0.3)

**0.4**	0.9(0.77)	97.95(3.25)	0.85(0.91)	0.05(0.22)

Total number of genes: 708. Number of differentially correlated genes: 100. Method: group method. Extended Bonferroni threshold: 1.0.

**Table 3:** SIMU1, true positives (TP) and false positives (FP) in simulations with independent base.

**Effect Size**	**CV Method**		**WRS Method**	
		
	FP mean(STD)	TP mean(STD)	FP mean(STD)	TP mean(STD)
**0.1**	0.95(0.92)	4.1(2.68)	0.65(0.85)	0.25(0.54)

**0.2**	0.65(0.85)	42.9(13.86)	0.75(0.83)	0.1(0.3)

**0.3**	0.65(0.73)	92.1(8.28)	0.9(1.09)	0.1(0.3)

**0.4**	1.45(1.07)	99.3(1.82)	0.85(0.91)	0.05(0.22)

Total number of genes: 708. Number of differentially correlated genes: 100. Method: resampling method. Extended Bonferroni threshold: 1.0.

**Table 4:** SIMU2, true positives (TP) and false positives (FP) in simulations with dependent base.

**Effect Size**	**CV Method**		**WRS Method**	
		
	FP mean(STD)	TP mean(STD)	FP mean(STD)	TP mean(STD)
**0.1**	1.05(2.77)	0.25(0.7)	0.05(0.22)	0.0(0.0)

**0.2**	0.85(2.87)	1.35(4.99)	0.15(0.48)	0.05(0.22)

**0.3**	0.55(1.56)	6.25(12.32)	0.15(0.48)	0.2(0.87)

**0.4**	1.1(4.57)	86.7(21.86)	0.65(1.35)	0.0(0.0)

Total number of genes: 708. Number of differentially correlated genes: 100. Method: resampling method. Extended Bonferroni threshold: 1.0.

For the resampling method, the computation time was 216 hours instead. All computations were done in a Saturn cluster computer which includes 2 nodes each with 8× AMD Opteron dual-core processors 2 GHz (16 processor cores), 16×2GB SDRAM.

Throughout this paper, we use the group method unless otherwise noted.

#### *N*-statistic

We chose a multivariate nonparametric test based on *N*-statistic with Euclidean kernel for testing the hypothesis ${H^{\prime }}_{i}$. This statistic has been successfully used to select differentially expressed genes and gene combinations in microarray data analysis [7-10]. The *N*-statistic is defined as follows:

(4) $$\begin{array}{lll} N_{i} & = & \frac{2}{n_{s}^{2}}\sum_{k=1}^{n_{s}}\sum_{l=1}^{n_{s}}L(w_{i}(A,k),w_{i}(B,l))-\frac{1}{n_{s}^{2}}\sum_{k=1}^{n_{s}}\sum_{l=1}^{n_{s}}L(w_{i}(A,k),w_{i}(A,l))\\ & & -\frac{1}{n_{s}^{2}}\sum_{k=1}^{n_{s}}\sum_{l=1}^{n_{s}}L(w_{i}(B,k),w_{i}(B,l)), \end{array}$$

where *n*_*s *_is the number of correlation vector samples in each group, **w**_*i*_(·, *k*) indicates the *k*th replication of the correlation vector (using the group or the resampling method), and $L(x,y)=||x-y||=\sqrt{\sum_{s=1}^{d}{\left(x_{s}-y_{s}\right)}^{2}}$ is the kernel defined by Euclidean distance.

After this step, the *i*th gene was assigned a non-negative number *N*_*i*_, a measurement of how much intergene correlation structure had changed from condition *A *to condition *B*.

#### 0.1 Resampling based *p*-values

We used the following algorithm to obtain resampling based *p*-values for each gene:

1. Randomly shuffle the slides in two different conditions, then split them into two groups.

2. Compute correlation vectors for each gene by using the group method or the resampling method.

3. Compute *N*-statistic for each gene based on the correlation vectors.

4. Repeat the above steps for *K *= 10, 000 times, record the resampling based *N*-statistics as *N*_*ik*_*, i *= 1, ..., *m*, *k *= 1, ..., *K*. They can be used to construct the (resampling based) null distribution for each index *i*.

5. Compute *N*_*i*_, the *N*-statistic for each gene (without random shuffles).

6. Obtain the resampling based *p*-value, *p*_*i*_, by comparing *N*_*i *_with the null distribution constructed from *N*_*ik*_. Specifically, *p*_*i *_is defined to be $\frac{\#(N_{ik}⩾N_{i})}{K}$, the proportion of *N*_*ik *_which is greater than or equal to *N*_*i*_.

Finally, we applied the extended Bonferroni adjustment [11] with threshold 1.0 to control PFER (per-family error rate). Extended Bonferroni adjustment is less conservative than the FWER (familywise error rate) controlling procedures and more stable than FDR (false discovery rate) controlling procedures in the context of microarray analysis. More details about this multiple testing adjustment procedure can be found in [11].

**Figure 1:**
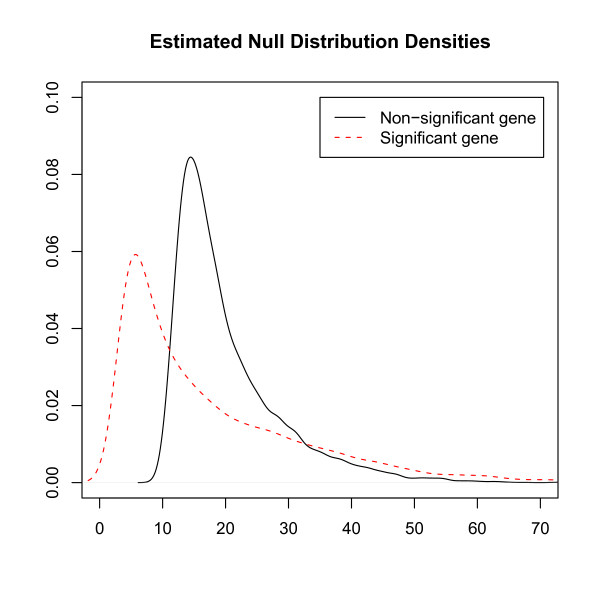
**Estimated Null Density Functions (resampling method with Bonferroni threshold 0.05) **without shuffling slides, the estimated N-statistic is 173.83 for the significant gene and 25.74 for the non-significant gene. The estimated density functions of most other genes follow the same pattern.

From the computational perspective, it was very tempting to reduce the number of permutations by pooling all *N*_*ik *_to construct one grand null distribution. However, we noticed that the null distributions for different genes can be very different. Based on our biological data, the density functions for the significant genes tended to shift to the left compared to those associated with the non-significant genes (see Figure 1).

### Univariate gene selection method

We would like to emphasize that our method (henceforward denoted as the **CV **method) is nonparametric. Because of this, we decided to compare the **CV **method to a nonparametrc univariate gene selection method: Wilcoxon rank-sum test with the same extended Bonferroni adjustment (henceforward denoted as the **WRS **method).

## Results

### Simulations

To gain better insight into the performance of the **CV **method, we simulated several sets of data. All sets had two groups of 80 arrays representing two different biological conditions (condition A and condition B). Each array had *m *= 708 genes. Denote the genes in the first condition by *x*_*i*_, 1 ≤ *i *≤ *m *and genes in the second condition by *y*_*i*_, 1 ≤ *i *≤ *m*. For both groups, all genes were identically distributed with marginal distribution *N*(0, 1). With different baseline correlation structure, we had the following two classes of simulated datasets:

**• SIMU1**: Any two distinct genes that were both in the set of the first 100 genes were correlated with coefficient *ρ*_*d *_in condition A, 0.0 in condition B. Otherwise the correlation coefficient was 0.0. Here *ρ*_*d *_was a constant taking value in {0.1, 0.2, 0.3, 0.4}. Condition B can be thought of as the control condition where genes were independent of each other. We called this dataset the independent base data.

**• SIMU2**: Any two distinct genes that were both in the set of the first 100 genes were correlated with coefficient 0.5 + *ρ*_*d *_in condition A, 0.5 in condition B. Otherwise, the correlation coefficient was 0.5. Again, *ρ*_*d *_was a constant taking value in {0.1, 0.2, 0.3, 0.4}. Unlike **SIMU1**, the baseline intergene correlation was 0.5. We called this dataset the dependent base data.

By this design, the differentially correlated genes were the first 100 genes for both **SIMU1 **and **SIMU2**. *ρ*_*d *_can be seen as a parameter indicating how much correlation structure had changed across two conditions.

For **SIMU1 **and **SIMU2 **and every *ρ*_*d*_, we applied both the **CV **method and **WRS **method, and recorded the true/false positives. We also repeated this process 20 times with different random seeds to get the mean and standard deviation of the true/false positives. The results are shown in Table 1 and Table 2. As expected, the **CV **method detected differentially correlated genes while the **WRS **method did not. The power of the **CV **method clearly increased as the effect size gets larger. Also, it was easier to detect differentially correlated genes in the independent base data than in the dependent base data. This means that high baseline correlation structure deteriorated the power of the **CV **method.

### Simulations with biological data

The difference between **SIMU1 **and **SIMU2 **was that the baseline intergene correlation was much higher in **SIMU2**. This was an attempt to model the intergene dependence structure in biological data. In some sense, a better way of modeling the actual dependence structure is through resampling from the biological data.

First, we combined **HYPERDIP **and **TEL **data and randomly permuted the slides. We then divided them into two groups of an equal number of slides, mimicking two biological conditions without differentially correlated genes. For both conditions, genes were standardized so that the sample means equaled zero and the sample standard deviations were one. We denoted the entries in two groups by *x*_*ij *_and *y*_*ij*_, 1 ≤ *i *≤ 7084 and 1 ≤ *j *≤ 79, and the correlation matrix of these two groups by {*ρ*_*ik*_}, 1 ≤ *i*, *k *≤ 7084.

Next, we generated a 79-dimensional random vector with *i.i.d*. standard normal components. Denote this vector by **a **= {*a*_*j*_}, 1 ≤ *j *≤ 79. We added **a **to the first 300 row vectors in the first condition with a tuning parameter *ρ *as follows: $\sqrt{1-\rho }x_{ij}+\sqrt{\rho }a_{j}$. These transformed entries are denoted as *x*_*ij *_again, and we name this dataset **SIMU3**.

The first condition had the correlation coefficients as follows:

$$corr(x_{i},x_{k})=\{\begin{array}{ll} \rho_{ik}+\rho (1-\rho_{ik}) & \text{for }1⩽i,k⩽300\text{ and }i\neq k,\\ \rho_{ik}\sqrt{1-\rho } & \text{for }1⩽i⩽300\text{ and }301⩽k⩽7084,\\ \rho_{ik} & \text{for }301⩽i,k⩽7084\text{ and }i\neq k, \end{array}$$

Noticeably, the correlation coefficients between any two of the first 300 genes of the first group differed substantially from those of the second group, and these were considered as differentially correlated genes. The correlation coefficients between any two of the remaining 6784 genes of the first group were the same as the corresponding correlation coefficients between those of the second group, so they were not considered as differentially correlated genes.

There was one caveat in this approach. Even if *x*_*i *_was one of the 6784 genes that were not differentially correlated, *corr*(*x*_*i*_, *x*_*k*_) was still different in two conditions if *x*_*k *_happened to be a gene from the first 300 genes. In other words, the first 300 differentially correlated genes induced some small changes for those genes that were not differentially correlated. In practice however, when summarized through the *N*-statistic, these differences for the latter 6784 genes were negligible and it was reasonable to view them as random fluctuations.

We set *ρ *= 0.5 in **SIMU3**. As before, we applied both the **CV **method and the **WRS **method. The above procedure was repeated 10 times, the results were summarized in Table 5.

**Table 5:** SIMU3, true positives (TP) and false positives (FP) in simulations of biological data with tuning parameter *ρ *= 0.5.

**CV Method**		**WRS Method**	
	
FP mean(STD)	TP mean(STD)	FP mean(STD)	TP mean(STD)
0.0(0.0)	270.6(11.09)	0.2(0.6)	0.0(0.0)

Total number of genes: 7084. Number of differentially correlated genes: 300. Method: group method. Extended Bonferroni threshold: 1.0.

The **CV **method selected most of differentially correlated genes while **WRS **method did not. The **CV **method also produced fewer false positives than the **WRS **method.

### Data analysis based on biological data

Using the **WRS **method, we found 102 differentially expressed genes. Using the **CV **method (resampling method was used here to gain more power), we detected 16 differentially correlated genes. Out of these, 11 were differentially expressed and 5 were not. These 5 genes were: CD1C (antigen precursor), HDHD1A (haloacid dehalogenase-like hydrolase domain containing 1A, enzyme involved in many catalytic activities), BASP1 (brain acid-soluble protein 1), CYB5A (cytochrome b5 type A, microsomal) and TFPI (tissue factor pathway inhibitor, which helps to regulate the extrinsic blood coagulation cascade). In the original study, two of these genes were found as differentiating among two leukemia subtypes (BASP1 and CYB5A) and the other three were never mentioned. Differential correlation of these genes in two leukemia subtypes might provide some valuable information for better understanding the underlying subtypes' differences; however, these genes could not be captured by conventional tests. The results of this study (with multiple thresholds before and after the Bonferroni adjustments) can be found in Table 6.

**Table 6:** Numbers of differentially expressed (DE) and differentially correlated (DC) genes from biological data before and after Bonferroni adjustment with variant significant levels.

	**Before Adjustment**	**After Adjustment**		
		
	level = 0.05	level = 0.05	level = 0.5	level = 1.0
**DC Genes**	275	10	10	16

**DE Genes**	421	68	93	102

**Both DC and DE Genes**	140	8	8	11

**DC But Not DE Genes**	135	2	2	5

**DE But Not DC Genes**	281	60	85	91

Total number of genes: 7084. Method: resampling method.

## Discussion and Conclusion

Our method represents a radical conceptual change from current approaches focused solely on differentially expressed genes. However, this method is not intended to replace the existing methodology but rather to provide biologists with an *additional source of information *for decision making. As an example, the univariate method failed to detect Gene CYB5A, which had a modest unadjusted *p*-value 0.168 based on Wilcoxon ranksum statistic. Yet CYB5A was detected as a differentially correlated gene with an unadjusted *p*-value 0.0 (its observed *N*-statistic was larger than all permutation *N*-statistics). To get a rough idea of how many genes had different correlation coefficients with CYB5A across two conditions, we looked at the marignal distributions of CYB5A' correlation vector. 252 genes were detected to have changed correlations with CYB5A dramatically across conditions. The selection procedure of these 252 genes can be summarized as follows: First, we splitted the slides in two conditions into 8 subgroups, respectively, as in the Group method. Second, we calculated 8 correlation vector samples of gene CYB5A in each condition. Finally, for all 7083 correlations (with 8 samples in each condition), we applied Wilcoxon rank-sum test to each of them to obtain an unadjusted *p*-value. After extended Bonferroni adjustment, 252 genes were selected at significant level 2.0. According to BioCarta, about one third of all pathways associated with these 252 genes are related to cell cycle progression, cell division and control of centrosome duplication. **HYPERDIP **phenotype is characterized by the presence of more than 50 chromosomes. As a consequence, all pathways working for cellular maintenance and proliferation should be higly activated in **HYPERDIP **phenotype. The differential correlation of genes involved in pathways, related to cell proliferation between **HYPERDIP **and **TEL **seemed reasonable and might deserve future studies.

The microarray technology yields unique multidimensional information on the functioning of the whole genome machinery at the level of transcription so that much can be learned about relationships between genes and mechanisms by which the cell assigns tasks to different genes to maintain a specific function. It is unfortunate that such an advanced technology continues to be used as a simplistic screening tool with a focus on big differences between mean values of expression measurements. The true potential of microarray technology has yet to be unveiled. It is noteworthy that recent years have seen a growing interest in correlations between gene expression levels in statistical methodologies for microarray analysis [9,12-25]. The correlation coefficient has been used extensively as a measure of similarity in gene clustering since a seminal paper by Eisen et al. [26]

However, very few studies have examined the possibility of using the intergene correlation structure to find important genes that are linked to disease. One obstacle lies in the fact that there are *m *different sample means but $\frac{m(m-1)}{2}$ different sample correlation coefficients. It is much harder to catch the differences hidden in the correlation matrix that has much higher degrees of freedom (25, 087, 986 in our study). Furthermore, it is much more computationally intensive to compute the sample correlation coefficients than the sample means. Consequently, we could not afford to use more than 10, 000 permutations to get finer *p*-value estimation, and we are thus reluctant to recommend the slower permutation based resampling method to the biologists, despite the fact that we know this method is more powerful than the group method.

As illustrated by our study on biological data (with 79 slides in each condition) we were able to identify 102 genes, which changes the medians of their (univariate) distributions, yet only 16 genes were reported as differentially correlated (see Table 6). This seeming inadequacy of power was also shown in the simulation studies (Table 1, Table 2). This phenomenon might be subject to a number of explanations. It might be caused by the small sample size. Due to the nature of the **CV **method, its statistical perspective is to compare the distribution of *sample correlation coefficients *instead of expression levels in different biological conditions. With the group method, we splitted 79 slides into subgroups and computed the sample correlation vectors from each group. As a result, we only had eight sample correlation vectors for each condition. We could divide the data into more subgroups, and then there would be fewer slides per subgroup so that the sample correlation coefficient computed from each subgroup would be less accurate. This was a trade-off. With the resampling method, we had 20 correlation vectors. Having more than 20 resamplings would enhance the accuracy of the estimated *N*-statistics and improve the power; meanwhile, it would demand more computing time, making this another trade-off.

The choice of the Euclidean distance as the kernel for the *N*-statistics might be another culprit. The Euclidean distance kernel is a *generic *kernel that is invariant under any orthogonal transformation. In other words, it is symmetric and indifferent to all departure from the null distribution. A specifically designed kernel that is sensitive to the likely departure from the null distribution caused by the changes of correlation might significantly increase the selection power of the **CV **method. Last, it may have been that indeed fewer genes were differentially correlated than were differentially expressed in the biological data. The hypotheses that the **CV **method was testing were entirely different from those tested by the univariate selection methods, such as the **WRS **method. It is absolutely possible that one gene is differentially expressed but not differentially correlated, or vice versa. The very fact that 11 out of 16 differentially correlated genes were differentially expressed in our study is an interesting phenomenon that is worth further investigation.

We believe many improvements can be made to enhance the selecting power of the **CV **method. We also firmly believe, as larger sets of microarray gene expression data become readily available, quantitative insights into dependencies between gene expression levels will gain increasing importance.

## Authors' contributions

The basic idea was first proposed by AY, LK and XQ. The detailed study design was developed by all members in the research team. RH carried out the needed computations and simulations and the majority of the software development. GG conducted the pathway analysis for differentially expressed and differentially correlated genes. RH and XQ were responsible for most of the write-up of the findings.
